# DNA Logic Gates for Small Molecule Activation Circuits in Cells

**DOI:** 10.1021/acssynbio.3c00474

**Published:** 2024-02-02

**Authors:** Cole Emanuelson, Anirban Bardhan, Alexander Deiters

**Affiliations:** Department of Chemistry, University of Pittsburgh, Pittsburgh, Pennsylvania 15260, United States

**Keywords:** nucleic acids, small molecule activation, DNA computing, DNA-templated chemistry

## Abstract

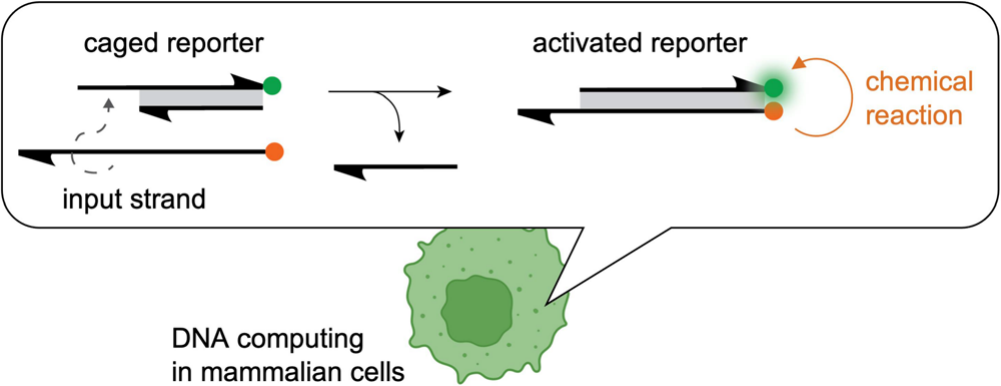

DNA-based devices such as DNA logic gates self-assemble into supramolecular structures, as dictated by the sequences of the constituent oligonucleotides and their predictable Watson–Crick base pairing interactions. The programmable nature of DNA-based devices permits the design and implementation of DNA circuits that interact in a dynamic and sequential manner capable of spatially arranging disparate DNA species. Here, we report the application of an activatable fluorescence reporter based on a proximity-driven inverse electron demand Diels–Alder (IEDDA) reaction and its robust integration with DNA strand displacement circuits. In response to specific DNA input patterns, sequential strand displacement reactions are initiated and culminate in the hybridization of two modified DNA strands carrying probes capable of undergoing an IEDDA reaction between a vinyl-ether-caged fluorophore and its reactive partner tetrazine, leading to the activation of fluorescence. This approach provides a major advantage for DNA computing in mammalian cells since circuit degradation does not induce fluorescence, in contrast to traditional fluorophore-quencher designs. We demonstrate the robustness and sensitivity of the reporter by testing its ability to serve as a readout for DNA logic circuits of varying complexity inside cells.

## Introduction

In the decades since the inception of the field of DNA nanotechnology in the 1980s, continual advancements have been made in the number and complexity of applications that utilize DNA to assemble nanostructures with a predefined structure and function.^1−4^ A subset of these advancements include those related to DNA computation, the use of DNA complexes that undergo highly dynamic reaction cascades to compute an output from one or more inputs.^2,5,6^

DNA logic gates provide a scalable platform for the assembly of complex circuits and devices. Individually, they are implemented as a series of toehold-mediated strand displacement reactions between input oligonucleotides and semistable multistrand gate complexes.^7,8^ One class of such gates is called “translator”, which converts one or more oligonucleotide inputs into an output oligonucleotide. This output may propagate through additional translator gates or serve as an input to a reporter gate, which is commonly implemented as double-stranded DNA modified with a fluorophore-quencher (FQ) pair. By layering and connecting multiple translator gates, Boolean logic operations (AND, OR, and NOT) can be performed.^7−9^ These logic gates can then be assembled into complex DNA circuits that detect a variety of input patterns and carry out sophisticated computations, including neural networks,^10^ pattern generation,^11^ execution of 6-bit algorithms,^12^ and data storage.^13^

While the field of DNA computation has undergone significant maturation since the first reported examples of algorithmic processing using DNA,^14,15^ the increase in the complexity of DNA circuits designed and executed within the controlled environment of a test tube has not translated directly into nucleic acid assemblies capable of operating within a living cell. The development of such biocompatible DNA-based devices has been a longstanding goal of DNA nanotechnology.^16^ The ability to predict the structure and dynamics of synthetic DNA circuits inspires the development of devices that directly interact with endogenous nucleic acids or other biomolecules to function as smart therapeutics or drug delivery vehicles.^17,18^ However, the capability and complexity of existing cellular DNA computation devices are more limited, commonly consisting of a reporter of a single input^19,20^ or circuit that computes a single layer of logic (e.g., AND or OR).^21−24^ Our group has aimed to expand the capabilities of DNA computation devices through the utilization of DNA-templated synthesis.^25,26^ The induced proximity that results from complementary oligonucleotide hybridization has been used to promote chemical reactions for a diverse set of applications including, drug release,^27−29^ RNA imaging,^30^ and small molecule activation.^31−34^ Our initial design, inspired by this field of work, featured DNA logic circuits that promote a Staudinger reduction between phosphine-modified and azido-modified DNA strands to release small molecule fluorophores.^34^ However, a drawback of this design, encountered during development, was the susceptibility of the phosphine to oxidation, which resulted in its deactivation and precluded gel purification and use in biological environments. Previous reports demonstrating DNA-templated Staudinger reactions have highlighted the oxidation issue of phosphine-modified oligonucleotides and have attempted to reduce the impact on functionality through the use of an excess phosphine probe.^29,35,36^ This limitation prompted us to investigate potential second-generation designs with more robust and physiologically stable reactive pairs, capable of withstanding purification, enabling the use of stoichiometric amounts of probes and, most importantly, operation in living cells.

## Results and Discussion

**Figure 1:**
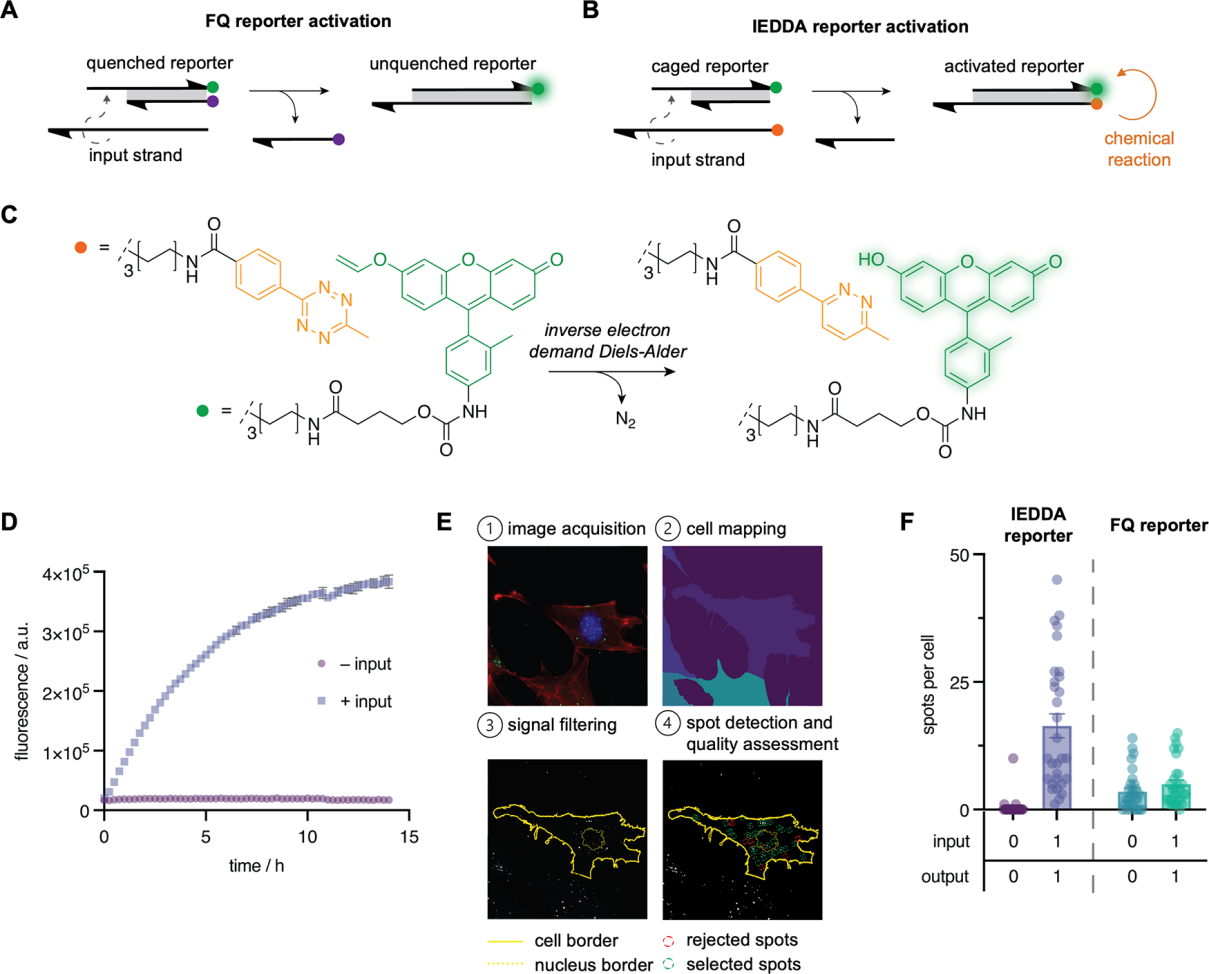
Concept and characterization of a DNA-templated IEDDA reaction for small molecule activation. (A) Strand displacement schemes for the FQ and (B) IEDDA reporter gates. (C) Reaction scheme of the proximity-induced IEDDA reaction between methyl-tetrazine (orange dot) and the vinyl-ether-caged fluorescein (green dot). (D) In vitro fluorescence time course activation of the caged reporter in presence and absence of input DNA. Mean fluorescence is shown ± s.d. *n* = 3. (E) Workflow for *in cellulo* detection of fluorescence activation. Full Z-stack images of cells transfected with caged reporter with and without input were fixed and stained with nuclei and actin dyes followed by cellular mapping, fluorescence filtering, and finally spot detection. (F) Quantification of the *in cellulo* for the IEDDA and FRET-based FQ reporters. The mean number of spots per cells is shown ± s.e.m. *n* = 30.

Here, we report a next-generation DNA-templated fluorophore activation reporter that utilizes an inverse electron demand Diels–Alder (IEDDA) reaction.^37^ This design was found to be stable during purification and to be capable of functioning within living cells. As such, the IEDDA reporter represents an alternative to the commonly employed FQ reporter where the dark state of the reporter is maintained only while the FRET pair remains in close proximity (Figure 1A). A significant drawback of FRET-based reporters for DNA strand displacement reactions is that they are prone to background activation due to unintended duplex separation, which may occur as the result of spontaneous dissociation of DNA base pairs, spurious displacement through interaction with partially complementary DNA, or enzymatic/chemical degradation of the DNA.^38,39^ Degradation poses a significant challenge to the application of DNA devices in biological environments, where enzymatic processing of FRET probes by endogenous nucleases can prevent robust signal detection. Our IEDDA reporter is less susceptible to background activation caused by nonspecific DNA interactions and leakiness because the transition from a dark OFF state to a fluorescent ON state is dependent on the induced proximity of reactant probes in a nontransient and specific fashion, as shown in Figure 1B, which, in contrast to FRET-pair separation, is unlikely to be facilitated by or occur as the direct result of enzymatic degradation. More specifically, fluorescence activation results from the removal of a vinyl-ether caging group from a caged fluorophore that occurs as a result of a proximity-induced reaction with a tetrazine probe (Figure 1C). In this study, probes were selected after careful consideration of the reactivity and stability during the design process. Importantly, the methyl-tetrazine and the vinyl-ether-caged fluorescein derivative are unreactive unless they are brought into close proximity (e.g., by DNA hybridization).^40^ Additionally, unlike other IEDDA substrates, such as strained *trans*-cyclooctene derivatives that can undergo isomerization to the *cis*-isomer within biological environments,^41^ the vinyl-ether moiety is stable. Synthesis of the vinyl-ether-caged fluorescein was adapted from a previous protocol (Scheme S1).^40^ The methyl-tetrazine reaction partner, which was synthesized, has been shown to be stable under physiological conditions.^42^

We first designed and synthesized a strand displacement reporter gate where the final state of DNA assembly places the reactive tetrazine and vinyl-ether-caged fluorophore in close proximity at the termini of a DNA duplex. The modified strands were synthesized through conjugation of the corresponding NHS esters to the terminally amino-modified strands (Scheme S2) and purified using high-performance liquid chromatography (HPLC) (Figure S1). Incubation of the vinyl-ether-caged fluorescein-modified DNA strand with an increasing concentration of the methyl-tetrazine small molecule (up to 1000 equiv.) did not result in any fluorescence activation (Figure S2). This observation confirms the reported limited reactivity of these two moieties in the absence of induced proximity. We next evaluated the potential to achieve templated activation of the caged reporter using an *in vitro* fluorescence assay to monitor fluorescence intensity with and without the addition of the tetrazine-modified input DNA (Figure 1D). The observed increase in fluorescence with the addition of input confirmed successful templated activation. The tetrazine-modified strand contains additional bases at the 3′ end relative to the toehold strand of the reporter. We tested the impact of these additional bases in the context of activation of the FQ reporter and observed similar activation in the presence of both inputs (Figure S3). Next, we evaluated the potential to achieve a high signal-to-background using this reporter, even under enzymatic DNA degradation conditions. Thus, the amount of fluorescence activation of our new IEDDA-based reporter and the traditional quencher-based reporter was measured in the presence of “DNase in TE-Mg^2+^ buffer, as well as in serum-containing cell culture media (Figure S4). Consistent with the described drawbacks of a FRET-based reporter, a significant increase in fluorescence was observed upon incubation of the FQ reporter with DNase and in Dulbecco’s modified Eagle's medium (DMEM) without the addition of input DNA. In contrast, the IEDDA reporter exhibits no background activation and exhibits an increase in fluorescence in the presence of input DNA, despite enzymatic degradation, as confirmed by gel electrophoresis (Figure S5).

Having confirmed the successful operation of the reporter gate, we next investigated the robustness of its activation in mammalian cells. As such, we adopted an imaging cytometry workflow to quantitatively assess the amount of activation and the signal-to-noise ratio of templated activation (Figure 1E). Briefly, cells were transfected with independently encapsulated reporter duplex and input oligonucleotides, following transfection cells were fixed and stained with dyes for actin (rhodamine phalloidin) and nuclear DNA (Hoechst 33342). To validate this assay, we confirmed that these separately encapsulated DNA complexes do not cross-react outside of the cell, confirming that the observed fluorescence is the result of reporter activation via strand displacement reaction within the cell (Figure S6). After fixation, the entire cell volume was imaged using a 63× objective to collect images spanning 6.5 μm in z-distance. From these images, a maximum intensity projection was then generated and processed using a MATLAB plugin called FISH-quant,^43^ which utilizes a series of filtering and denoising algorithms to identify and assess the quality of fluorescent puncta present in high content imaging data sets. Developed for the processing of RNA fluorescence in situ hybridization data sets, this program has been utilized in other applications of cellular fluorescence microscopy for the detection and quantification of diffraction-limited spots emitted from fluorescent probes targeted toward DNA–protein,^44^ RNA–protein,^19,45^ and RNA–RNA interactions.^46,47^ Here, we have applied this program to quantify the puncta resulting from input and reporter overlap within endocytic vesicles, as observed in other applications of intracellular strand displacement reporters^38^ and single-stranded fluorescently labeled oligonucleotides delivered through lipid nanoparticle transfection.^48^ We then used this workflow to compare the mean number of fluorescent spots observed per cell transfected with the new templated IEDDA reporter and the traditional FQ reporter with and without the corresponding input (Figure 1F). Intriguingly, the chemical reaction-based IEDDA reporter exhibited an average of 16.4 fluorescent spots per cell when cotransfected with input DNA and 0.40 spots without input. This increase is a significant advancement in signal-to-noise ratio over the FQ reporter, for which the mean spots per cell detected were 5.03 and 3.50 with and without the same input delivery, respectively, thereby validating our hypothesis of improved signal-to-background ratio of our current DNA computation reporter design.

**Figure 2:**
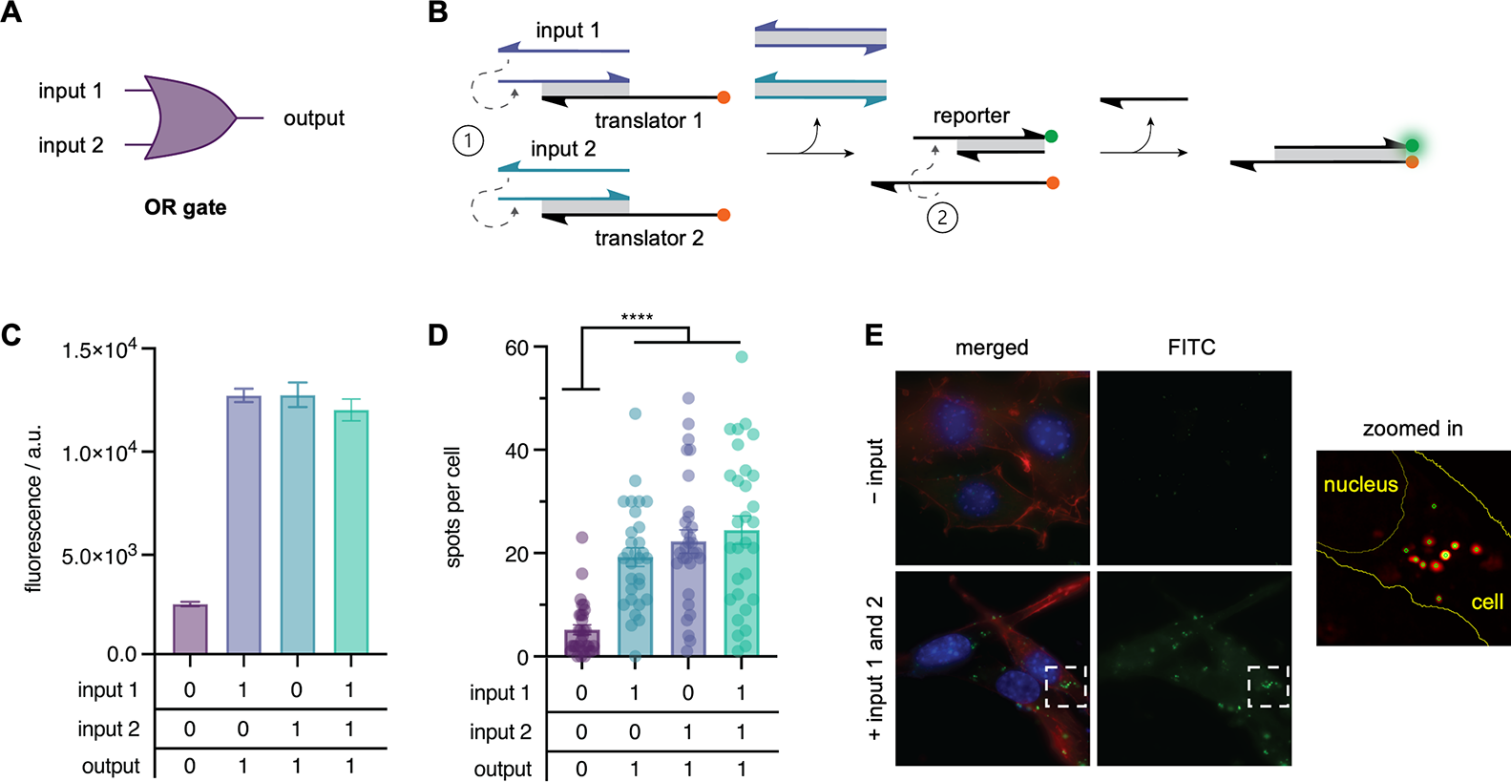
Templated OR circuit scheme and activation. (A) OR circuit diagram. (B) DNA strand displacement scheme resulting in the proximity-driven IEDDA reaction between methyl-tetrazine and vinyl-ether-caged fluorescein. (C) Mean fluorescence intensity of *in vitro* OR circuit activation (mean ± s.d.; *n* = 3). (D) Activation of the OR circuit in cells. Mean fluorescent spots per cell are shown (mean ± s.e.m.; *n* = 30). ****p < 0.0001; calculated from multiple unpaired two-tailed Student’s *t*-test. (E) Representative maximum intensity projections of cells transfected with reporter and translator gates with and without DNA inputs 1 and 2.

DNA strand displacement devices are uniquely suited for the design and implementation of programmable networks of interacting DNA complexes. By selecting sequences with tiered layers of complementarity and specific single-stranded toehold domains, it is possible to accurately anticipate the order of strand displacement reactions that will proceed in the presence of oligonucleotide inputs, allowing for the assembly of DNA-based Boolean logic gates into complex circuits.^7,8,22,49−51^ To test whether the observed improvement in the signal-to-background ratio for the IEDDA reporter would permit its application in more complex DNA strand displacement cascades, we synthesized and tested an OR gate (Figure 2A). The gate is composed of two double-stranded “translator” complexes, each of which releases a tetrazine-modified DNA strand in the presence of a specific input DNA. The modified strand released from either translator gate is complementary to the caged reporter such that it will hybridize with the reporter and facilitate the IEDDA reaction producing a fluorescent signal (Figure 2B). We first validated the OR circuit *ex cellulo*, by incubation of the caged reporter, translator 1, and translator 2 gates in the presence of either or both input DNAs (Figure 2C). An increase in fluorescence was observed upon incubation with either or both input 1 and input 2 DNA strands, replicating the expected output pattern of an OR gate. Having demonstrated expected OR gate behavior in an *in vitro* assay, we then assessed the activity of the system in cells using the same workflow as described above (Figure 1D) to quantify the amount of activation observed under each combination of input DNAs (Figures 2D,E and S7). The results of this *in cellulo* analysis were consistent with the activation pattern observed *in vitro*. Cells cotransfected with either or both input DNA strands showed significantly more fluorescent spots per cell than those transfected with only the reporter and translator components, representing an approximate fourfold increase over background. Encouragingly, a similar signal-to-background ratio was observed both *ex* and *in cellulo*, an observation that supported our hypothesis that insulating reporter activation from degradation would lead to an improvement in circuit performance in cells.

**Figure 3:**
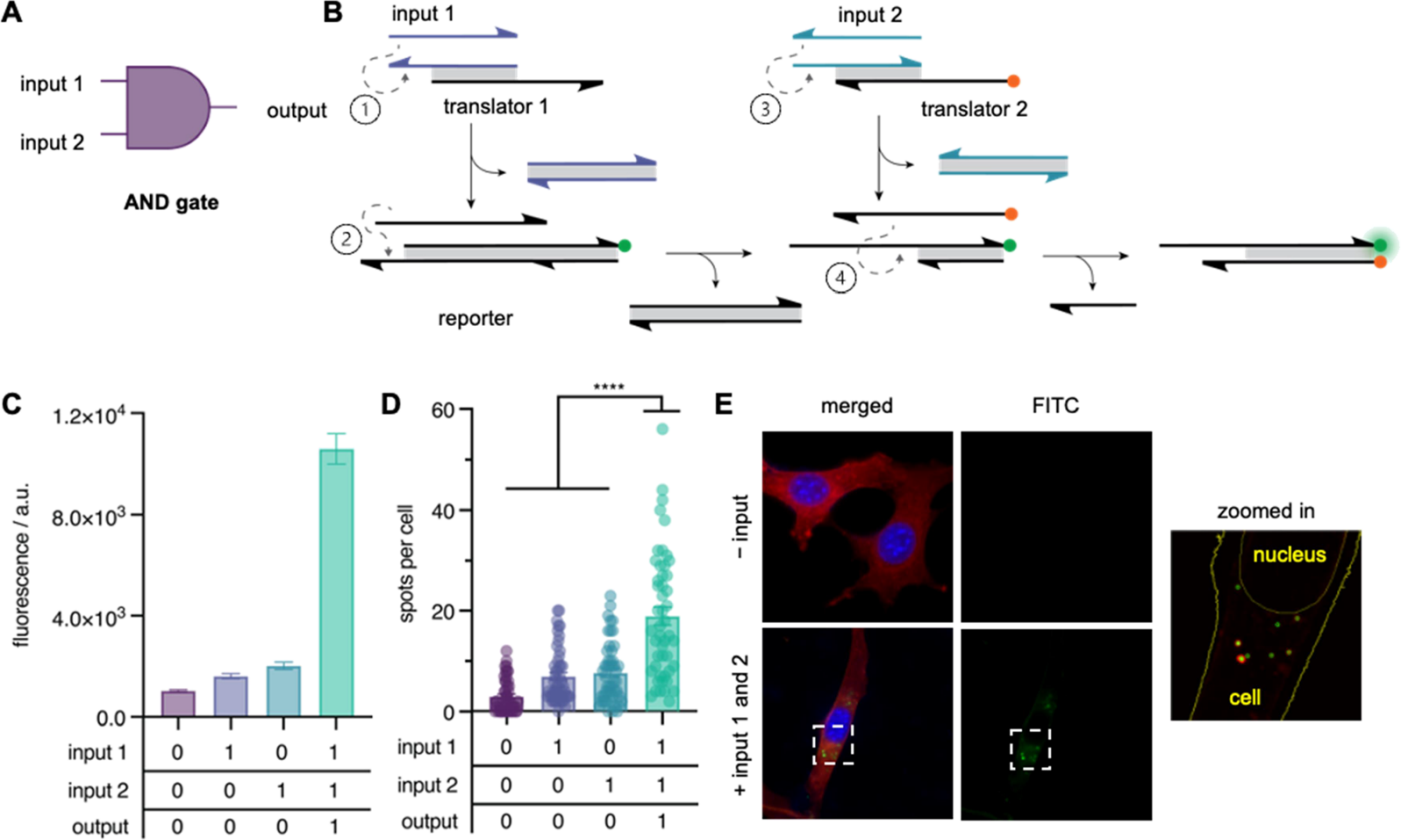
Templated AND circuit scheme and activation. (A) AND circuit diagram. (B) DNA strand displacement scheme resulting in the proximity-driven IEDDA reaction between methyl-tetrazine (orange dot) and vinyl-ether-caged fluorescein (green dot). (C) Mean fluorescence intensity of *in vitro* activation of the AND circuit (mean ± s.d.; *n* = 3). (D) Activation of the AND circuit in cells. Mean fluorescent spots per cell are shown (mean ± s.e.m.; *n* = 50). ****p < 0.0001; calculated from multiple unpaired two-tailed Student’s *t*-test. (E) Representative maximum intensity projections of cells transfected with reporter and translator gates with and without DNA inputs 1 and 2.

In addition to the OR logic gate described above, we sought to test the ability of our IEDDA reporter gate to interface with another fundamental logic gate, the AND gate (Figure 3A). While an OR gate produces a TRUE output in the presence of any accepted input, a TRUE output is produced from an AND gate only if all accepted inputs are present. The strand displacement scheme in Figure 3B depicts the series of strand displacement reactions in this circuit. Three upstream strand displacement reactions precede the templating hybridization reaction including input 1 with translator 1, the output from this reaction with the toehold strand of the reporter gate, and input 2 with translator 2. The strand displacement reaction between input 2 and translator 2 releases the tetrazine-modified DNA strand, which hybridizes to the exposed toehold on the reporter gate. This strand displacement circuit was first verified in an *ex cellulo* fluorescence assay, which showed that in the presence of inputs 1 and 2, a significant increase in fluorescence was observed, reproducing the expected output from an AND logic gate (Figure 3C). To further evaluate the performance of the circuit, cells were transfected with translator 1, translator 2, and reporter gates and each combination of input DNA strands. The quantified results from this experiment are shown in Figure 3D. As can be seen in the representative micrographs (Figures 3E and S8), cells transfected with both input 1 and input 2 exhibited a greater mean number of fluorescent spots per cell than those transfected with only a single input or without input DNA, with a minimum fold change of approximately 2.5 over the background. With this observation, we determined that the IEDDA reporter could be successfully implemented as a reporter for two fundamental logic gates inside cells.

**Figure 4:**
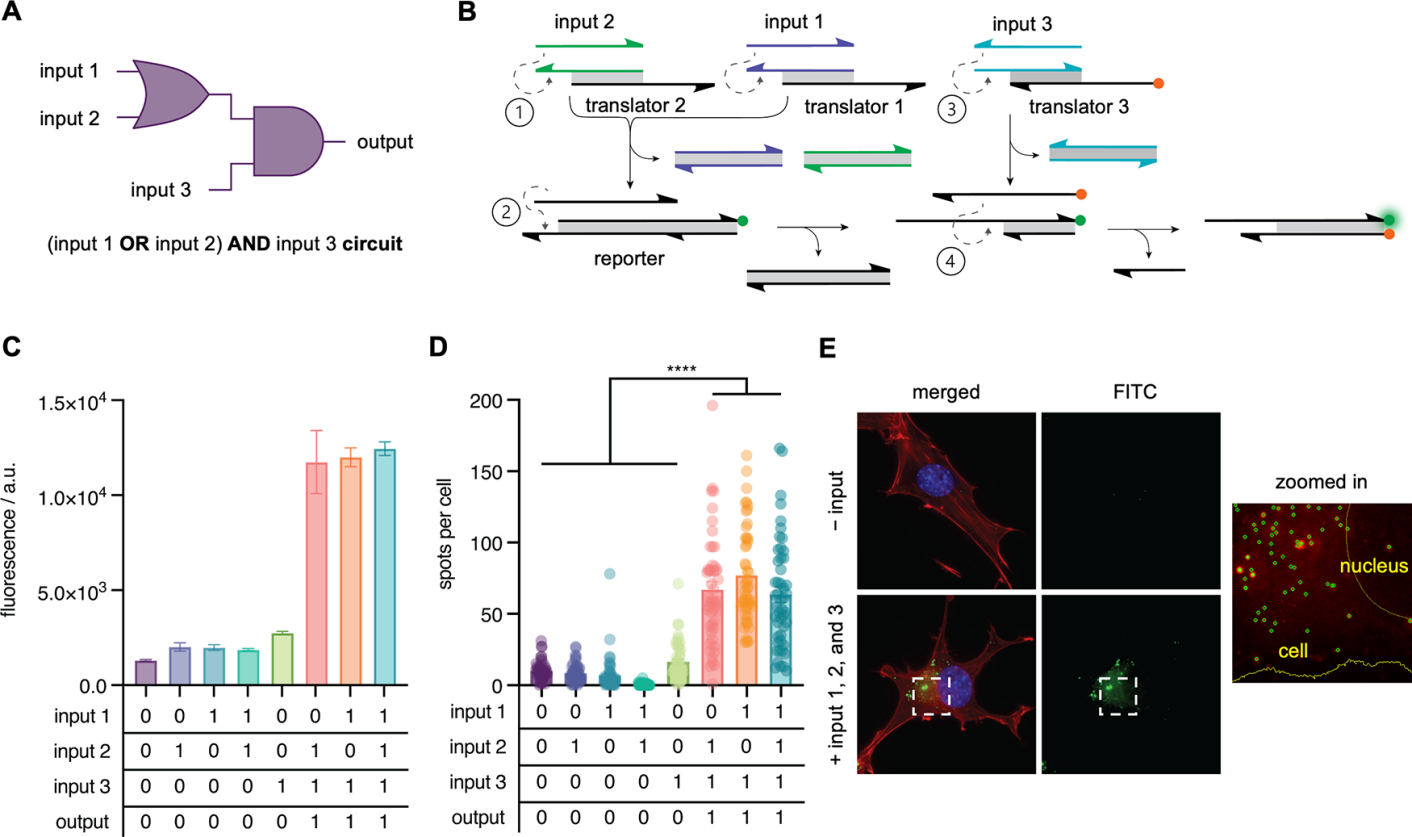
Templated multi-layer OR-AND circuit scheme and activation. (A) OR-AND circuit diagram. (B) DNA strand displacement scheme resulting in the proximity-driven IEDDA reaction between methyl-tetrazine and vinyl-ether-caged fluorescein. (C) Quantification of *in vitro* activation of the OR-AND circuit. Mean fluorescence intensity is shown after 12 h of circuit incubation (mean ± s.d.; *n* = 3). (D) Activation of the OR-AND circuit in cells. Mean fluorescent spots per cell are shown (mean ± s.e.m.; *n* = 50). ****p < 0.0001; calculated from multiple unpaired two-tailed Student’s *t*-test. (E) Representative maximum intensity projections of cells transfected with reporter and translator gates with and without DNA inputs 1, 2, and 3.

The DNA strand displacement circuits described above accept two distinct inputs, characteristic of the basic logic gates that these DNA circuits represent. As an extension of these fundamental gates, the combination of multiple Boolean logic gates into multilayered circuits provides a straightforward strategy to increase the complexity of the computation process through scaling the number of inputs processed by the DNA circuit. To evaluate the potential of the IEDDA reporter to integrate into such a multi-layer device, we designed and tested an OR-AND logic circuit that terminates in IEDDA reaction initiation (Figure 4A). The DNA strand displacement scheme shown in Figure 4B illustrates this circuit, which integrates three separate DNA inputs that interact with three translator gates and a single reporter gate. The individual steps are numbered to illustrate the sequence of strand displacement events necessary for reporter activation: One, input 1 or input 2 must be present to react with either translator 1 or 2. Two, a strand released from either translator 1 or 2 hybridizes with the exposed toehold on the reporter. Three, input 3 hybridizes with translator 3 and displaces the tetrazine-modified strand. Four, the tetrazine-modified strand hybridizes to the exposed toehold on the reporter gate. The circuit was first evaluated in an *ex cellulo* fluorescence assay, which revealed that incubation with each of the three input combinations expected to give a TRUE output in an OR-AND circuit yielded fluorescence intensity values greater than combinations where the expected output was FALSE (Figure 4C). Having confirmed the expected performance of the OR-AND circuit in an *in vitro* fluorescence assay, we sought to evaluate the activity of the circuit in cells by utilizing the same workflow described for the single-layer gates described above. Gratifyingly, the *in cellulo* activation pattern, as shown in Figure 4D, was found to correlate well with the *in vitro* results and the expected output of the represented circuit, with the lowest value for a TRUE input combination being approximately 4 times greater than the background. Representative micrographs of cells transfected with reporter and translator gates, with and without input DNA, are shown in Figure 4E and in Figure S9. The high number of fluorescent spots per cell observed for the TRUE outputs over the FALSE outputs highlighted the robustness of this IEDDA reporter in cells and the fact that it can be utilized to construct even more complex DNA circuits.

## Conclusions

In summary, we have designed, synthesized, and characterized the performance of a robust DNA-templated, reaction-based fluorescent reporter. Toward this end, we developed and prepared DNA strands terminally modified with proximity-induced IEDDA reactive probes that are sufficiently stable to undergo HPLC purification, DNA complex assembly, and purification, as well as transfection into mammalian cells. One reaction partner is a vinyl-ether fluorescein derivative, which exhibits diminished fluorescence due to the internal charge transfer.^40^ The other reaction partner is methyl-tetrazine, which is conjugated to a DNA input strand. Upon hybridization of the modified DNA strands, the vinyl-ether-caged fluorophore and tetrazine undergo a “click to release” event that proceeds through an IEDDA reaction, followed by an elimination step, which produces an activated fluorophore with a free hydroxyl group. Using this templated activation strategy, we developed a DNA strand displacement reporter that functions more robustly inside cells than a FRET-based reporter composed of identical DNA sequences. This more robust performance is attributed to the fact that enzymatic degradation of the IEDDA reporter does not produce a nonspecific increase in fluorescence, which is a source of background in FRET-based reporters. This design overcomes the limitations of our first-generation Staudinger reaction approach, which utilized a phosphine probe that was susceptible to oxidation and precluded *in cellulo* applications. Additionally, we demonstrated the reporter’s utility by designing and testing DNA strand displacement circuits that represent Boolean logic gates of single- and multi-layer complexity, through the detection of DNA inputs monitored by the change in fluorescence. These DNA-based logic circuits showed robust function upon transfection into mammalian cells due to reduced sensitivity to enzymatic DNA degradation and enhanced signal-to-background. The proximity-induced IEDDA reaction for the reporter gate activation demonstrates an alternative approach to the use of backbone-modified oligonucleotides for the design of reporter devices with improved stability for cellular applications.^38,52^ Sugar and phosphate modifications, such as 2’F, 2’OMe, phosphorothioate, peptide nucleic acids, and locked nucleic acids, not only increase the cost of complex circuits but also can introduce changes to the nucleic acid hybridization, dynamics, and interaction thereby hindering translation from DNA-based devices into cell-stable circuits. By applying templated chemistry, DNA-based devices that function independently of nuclease degradation can be designed and tested by using easily obtained synthetic oligonucleotides with standard DNA backbones.

## Experimental Section

### Logic Gate Preparation and Purification

DNA complexes were purified as previously reported.^49^ Briefly, gate duplexes were assembled at 20 μM in 100 μL of 1× TE/Mg^2+^ buffer (Tris-HCl [10 mM; pH 8.0], EDTA [1 mM], and MgCl_2_ [12.5 mM]) and annealed by cooling the solution from 95 to 12 °C over 30 min in a thermal cycler (Bio-Rad, T100). Detailed descriptions for the assembly of individual gate complexes are described in the Supporting Information. Gates were then purified on a 16% native polyacrylamide gel electrophoresis gel. The full-size duplex bands were identified using a hand-held UV lamp (4 W, Analytik Jena, UVL-21) via UV shadowing on a TLC plate, excised, cut into small pieces, and eluted overnight in 300 μL of TE/Mg^2+^ buffer. Gate concentrations were determined by UV absorption at 260 nm using a Nanodrop ND-1000 (Thermo Fisher Scientific) and calculated with the appropriate duplex extinction coefficient.

### Fluorescence Activation Measurement

Each reaction was prepared to a final volume of 50 μL in 1× TE/Mg^2+^ buffer in a 384-well flat black plate (Greiner). TAMRA fluorescence was measured on a TECAN INFINITE M1000 Pro microplate reader (ex/em 545/565 nm) for the indicated time. Fluorescence intensity across experimental conditions were analyzed and plotted using Prism 9 graphing software (GraphPad).

### Cell Culture

All cell culture experiments were performed in a sterile laminar flow hood. NIH 3T3 cells were maintained in DMEM (Gibco, SH30003.03) supplemented with 10% (v/v) fetal bovine serum (Sigma-Aldrich, F0926) and 1% (v/v) penicillin/streptomycin (Corning, 30002CI) at 37 °C with 5% CO2.

### Fluorescence Imaging and Quantification

NIH3T3 cells were seeded into an 18-well chamber slide with a glass coverslip (ibidi, 81817, 15,000 cells/well) in 100 μL of DMEM. Following overnight incubation, media was replaced with 70 μL of DMEM (antibiotic-free). Cells were transfected with the indicated combination of DNA reporter (50 nM), translator (1.25×), and input (2.5×), separately encapsulated in 10 μL of Opti-MEM transfection media, using FuGENE HD transfection reagent (Promega, E2311, 3:1 μL reagent: ng DNA ratio). Following 24 h incubation, cells were washed twice with 50 μL of phosphate-buffered saline (PBS), fixed by immersion in 50 μL of 4% formaldehyde in PBS for 10 min at room temperature, and washed twice more in PBS. Fixed cells were stained in a 50 μL of PBS solution containing 1× phalloidin rhodamine (ThermoFisher, R415) and DAPI (Invitrogen, D1306) F-actin and nuclei dyes, then washed twice and immersed in PBS. Whole-cell z-stack images (27 slices, 250 nm spacing) were obtained using SlideBook 6 imaging suite (3i) and a Zeiss Axio Observer Z1 with LED light source (X-Cite LED Boost), 63× oil immersion objective (Zeiss Plan-Apochromat), sCMOS camera (Andor Zyla 4.2), FITC (ex. 470/40, em. 525/50), TRITC (ex. 545/25, em. 605/70), and DAPI (ex. 395/25, em. 460/50) filter sets. Images were exported as TIFF files and processed using ImageJ (National Institute of Health). The number of fluorescent spots per cell in each image were quantified using FISH-quant.^43^

## Supporting Information Available

Detailed synthetic protocols, sequence information, and supporting figures (PDF)
