# Supplementary material for: DNA Logic Gates for Small Molecule Activation Circuits in Cells
Source: ACS Synth Biol. 2024 Feb 2;13(2):538–45. doi: 10.1021/acssynbio.3c00474 (PMC10877608; doi:10.1021/acssynbio.3c00474)
Supplement: Supplementary file 1 — sb3c00474_si_001.pdf [file sb3c00474_si_001.pdf]

Supporting Information

DNA Logic Gates for Small Molecule Activation Circuits in Cells

Cole Emanuelson‡, Anirban Bardhan‡, and Alexander Deiters*

University of Pittsburgh, Department of Chemistry, Pittsburgh, PA 15260

**To whom correspondence should be addressed: deiters@pitt.edu*

Table of Contents 1
Experimental methods and materials 2
General chemical methods 2
Synthesis of probes 2
Preparation of modified oligonucleotides 5
Supporting Figures 6
Supporting Table 13
Characterization Spectra 14
References 15

Experimental methods and materials

General chemical methods

All reagents were purchased from commercial suppliers and used without further purification. All reactions for the synthesis of the chemical probes were performed in flame-dried glassware and stirred magnetically. Flash chromatography was performed by hand. NMR spectra were recorded on Bruker Ultrashield 300 MHz, 400 MHz, or 500 MHz spectrometers (Billerica, MA). Analytical LC-MS data was collected on a Shimadzu LCMS-2020 (Santa Clara, CA) and ionized via electrospray ionization (ESI, $\pm$ve) method. High resolution mass spectrometry (HRMS) was performed by the University of Pittsburgh facilities using Thermo Scientific Q-Exactive Orbitrap (Waltham, MA) and ionized via electrospray ionization (ESI, $\pm$ve) method.

Synthesis of probes

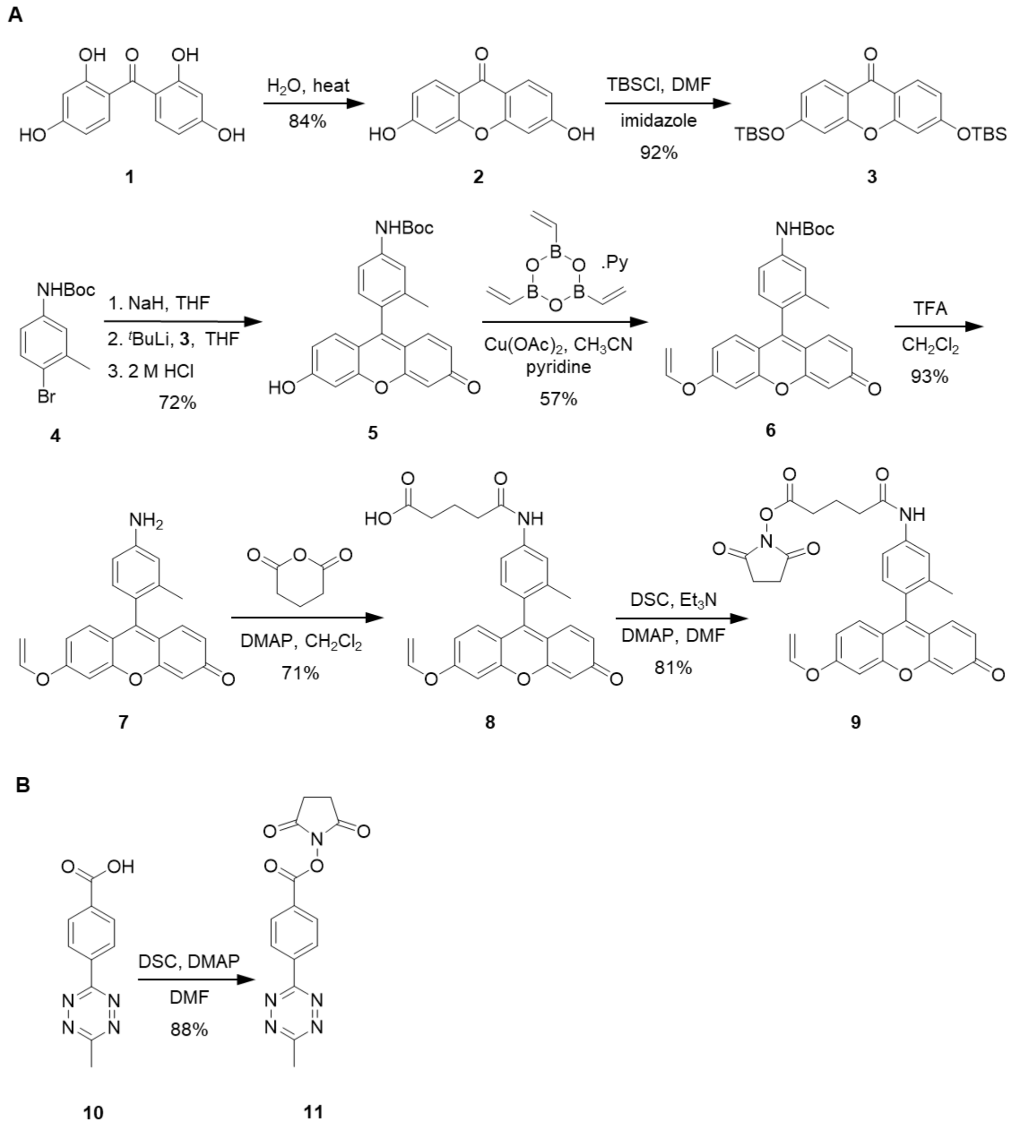

Supporting Scheme S1. Synthesis of probes used in this study. A) Synthesis of vinyl ether-caged fluorescein NHS ester **9**, and B) methyl-tetrazine NHS ester **11**.

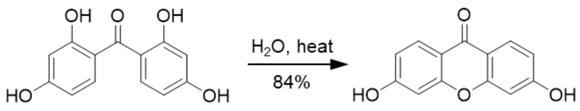

1 **2**

3,6-Dihydroxy-9H-xanthen-9-one (2). Xanthone **2** was synthesized from compound **1** following a previously established protocol and the analytical data matched reported results.[1]

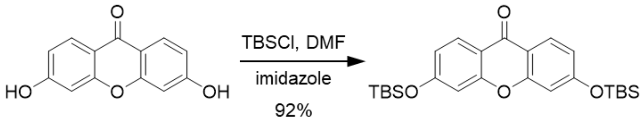

2 **3**

3,6-bis((*tert*-Butyldimethylsilyl)oxy)-9H-xanthen-9-one (3). Fluorescein xanthone **3** was synthesized from compound **2** following a previously established protocol and the analytical data matched reported results.[2]

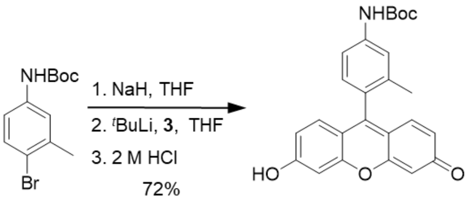

4 **5**

***tert*-Butyl (4-(6-hydroxy-3-oxo-9,9a-dihydro-3H-xanthen-9-yl)-3-methylphenyl)carbamate (5).** Fluorescein derivative **5** was synthesized from xanthone **2** following a previous report and the analytical data matched reported results.[3]

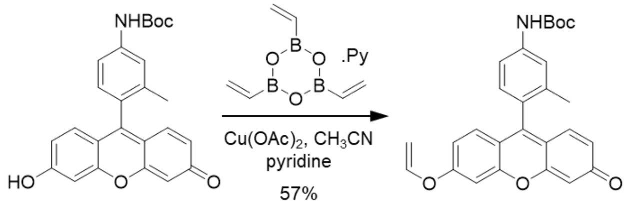

5 **6**

***tert*-Butyl (3-methyl-4-(3-oxo-6-(vinyloxy)-9,9a-dihydro-3H-xanthen-9-yl)phenyl)carbamate (6).** Copper (II) Acetate (26 mg, 0.14 mmol) was added to a flame-dried vial and dissolved in 2 mL of CH_3CN and was stirred at room temperature for 15 minutes. 2,4,6-Trivinylcyclotriboroxane-pyridine complex (27.4 mg, 0.11 mmol), compound **5** (60 mg, 0.14 mmol), and pyridine (0.12 mL, 14.3 mmol) were added to the reaction mixture. The mixture was then stirred at 40 °C for 36 h, followed by extraction with EtOAc (2 x 30 mL), washed with water (3 x 50 mL) and brine (50 mL) to remove salts and pyridine, before being dried over anhydrous Na_2SO_4 (1 g). The solvent was then removed in vacuo and the residue was purified by flash chromatography on silica gel, eluting with hexanes:EtOAc (1:2) to give the vinyl ether-caged fluorescein derivative **6** (36.2 mg, 57%) as an orange solid. The analytical data matched reported results.[3]

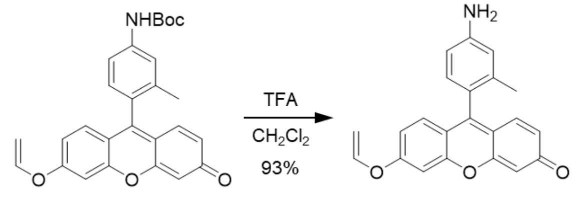

6 **7**

9-(4-Amino-2-methylphenyl)-6-(vinyloxy)-9,9a-dihydro-3H-xanthen-3-one (7). Vinyl ether-caged compound **6** (36.2 mg, 0.081 mmol) was dissolved in CH_2Cl_2 (3 mL) and cooled to 0 °C. Trifluoroacetic acid (0.33 mL; final concentration 10% v/v) was added to the solution and the mixture was stirred at 0 °C for 2 h after TLC showed complete spot to spot conversion of the starting material to the product. Then the reaction mixture was concentrated in vacuo and the crude product **7** (26.7 mg) was used directly for the next step without further purification. LCMS-ESI m/z calculated for $C_{22}H_{18}NO_3$ $[M+H]^+$: 344.12, observed 344.00.

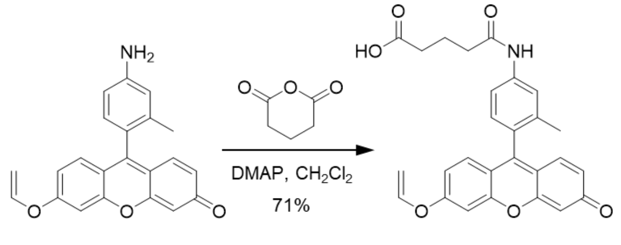

7 **8**

5-((3-Methyl-4-(3-oxo-6-(vinyloxy)-9,9a-dihydro-3H-xanthen-9-yl)phenyl)amino)-5-oxopentanoic acid (8). Compound **7** (26 mg, 0.075 mmol) was dissolved in CH_2Cl_2 (4 mL) and glutaric anhydride (17.2 mg, 0.15 mmol) and DMAP (18.4 mg, 0.15 mmol) were added. The reaction mixture was heated under reflux overnight before concentrating it *in vacuo*. The residue was purified by flash chromatography on silica gel, eluting with CH_2Cl_2:CH_3OH:AcOH (19:1:0.2) to give the vinyl ether-caged fluorescein acid **8** (24.4 mg, 71%) as an orange solid. ^1H NMR (500 MHz, CD_3OD) δ ppm 2.09 (s, 3 H) 2.15 (quin, *J* = 6.52 Hz, 2 H) 2.68 (s, 1 H) 2.86 - 2.91 (m, 4 H) 4.74 (dd, *J* = 5.95, 1.83 Hz, 1 H) 5.02 (dd, *J* = 13.43, 1.83 Hz, 1 H) 6.52 (d, *J* = 1.98 Hz, 1 H) 6.65 (dd, *J* = 9.61, 1.98 Hz, 1 H) 7.00 (dd, *J* = 13.43, 5.95 Hz, 1 H) 7.08 (dd, *J* = 9.00, 2.44 Hz, 1 H) 7.23 (dd, *J* = 8.09, 1.68 Hz, 1 H) 7.28 - 7.31 (m, 2 H) 7.32 - 7.38 (m, 3 H); HRMS-ESI m/z calculated for $C_{27}H_{25}NO_6$ $[M-H]^+$: 458.1609, observed 458.1953.

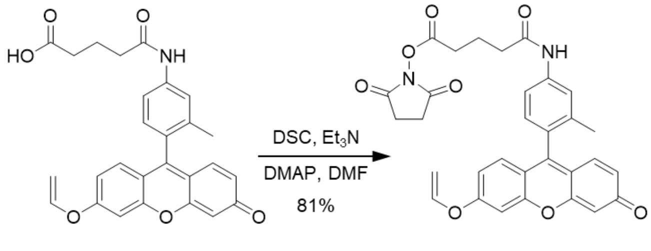

8 **9**

2,5-Dioxopyrrolidin-1-yl 5-((3-methyl-4-(3-oxo-6-(vinyloxy)-9,9a-dihydro-3H-xanthen-9-yl)phenyl)amino)-5-oxopentanoate (9). Vinyl ether-caged fluorescein acid **8** (10 mg, 0.02 mmol) was dissolved in DMF (2 mL) and the solution was stirred at room temperature. *N,N'*-Disuccinimidyl carbonate (11.2 mg, 0.04 mmol), triethylamine (8.5 µL, 0.06 mmol), and DMAP (1.5 mg, 0.01 mmol) were added and the mixture was left to stir overnight at room temperature. Next, the reaction mixture was concentrated *in vacuo* and purified by flash chromatography on silica gel, eluting with CH_2Cl_2:CH_3OH (49:1) to furnish the NHS ester **9** (9.8 mg, 81%). The analytical data matched reported results.[3]

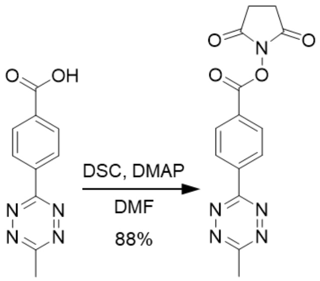

10 **11**

2,5-Dioxopyrrolidin-1-yl 4-(6-methyl-1,2,4,5-tetrazin-3-yl)benzoate (11). Compound **10** (15 mg, 0.07 mmol) was dissolved in DMF (2 mL) and to the solution was added *N,N'*-disuccinimidyl carbonate (36 mg, 0.14 mmol) and DMAP (21 mg, 0.18 mmol). The reaction mixture was left to stir overnight at room temperature following which the solvent was evaporated in vacuo. The residue was then purified by flash chromatography on silica gel, eluting with hexanes:EtOAc (1:4) to furnish the acetate **10** (19.2 mg, 88%). The analytical data matched previously reported results.[4]

Preparation of modified oligonucleotides

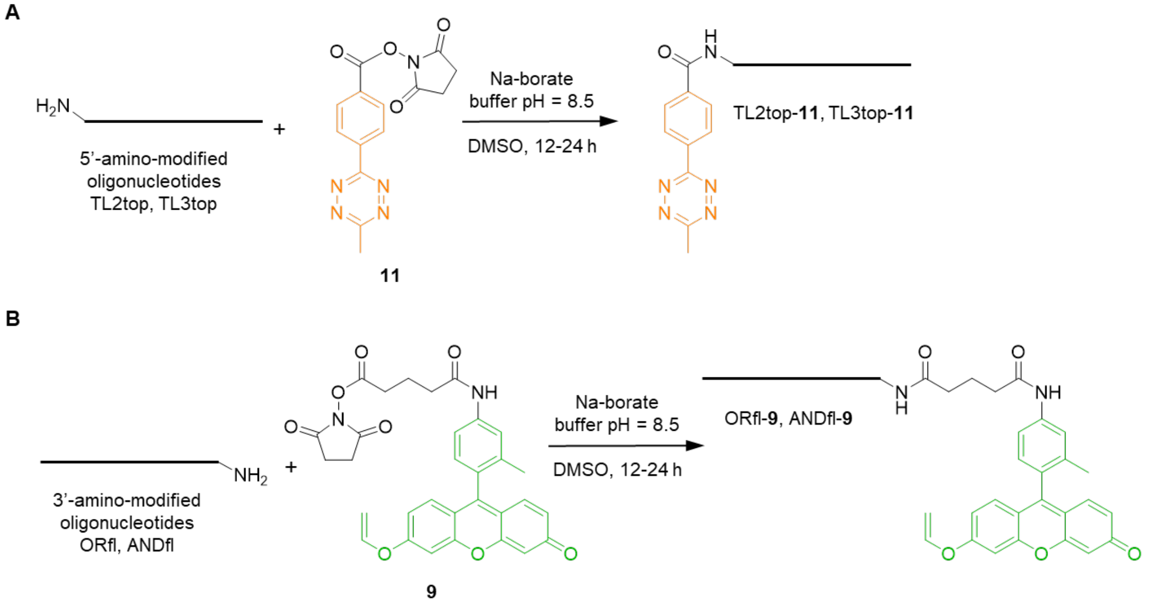

Supporting Scheme S2. Conjugation of probes to amino-modified oligonucleotides. A) Synthesis of 5'-methyl tetrazine conjugated oligonucleotides TL2top-**11** and TL3top-**11**. B) Synthesis of 3'-vinyl ether-caged fluorescein conjugated oligonucleotides ORfl-**9** and ANDfl-**9**.

All oligonucleotides were purchased from Integrated DNA Technologies, IDT (Coralville, IA) and were diluted to final stock concentration of either 100 µM in nuclease-free water and used directly without further purification (for sequence information see **Supporting Table 1** below). For generation of the modified oligonucleotides, 100 µL of the amino-modified oligonucleotides (100 µM; TL2top, TL3top, ORfl, ANDfl) were concentrated down and redissolved in 60 µL of Na-borate buffer (0.1 M; pH = 8.5). Then 25 µL (50 equiv.) of a solution of the corresponding NHS ester (**9** or **11**; 20 mM in DMSO) were added. DMSO (15 µL) was added to the reaction mixture to bring the final volume to 100 µL (40% final DMSO concentration).

The reaction mixture was left at room temperature for 12-24 h. After incubation, 10 µL of sodium acetate (3 M in water) were added followed by addition of 520 µL of EtOH. The mixture was cooled to –80 °C for 30 minutes to ensure complete oligonucleotide precipitation. The oligonucleotide was pelleted through centrifugation at 13,000 g (4 °C) for 10 min, and the supernatant was removed. Next, the pellet was washed with EtOH (2 x 400 µL) followed by 70% EtOH (2 x 400 µL), centrifuging each time for 10 minutes at 4 °C and removing the supernatant before proceeding with the next wash step. Following final centrifugation and removal of supernatant, the pellet was dissolved in 100 µL of water. The oligonucleotide was then analyzed/purified by HPLC (**Supporting Figure S1**) using an ACE Excel 3 Oligo Beta Test C18 column (4.6 mm x 100 mm, 1.7 µM). Triethylammonium acetate buffer (0.01 M, pH = 7.0) as used as solvent A and acetonitrile as solvent B. The gradient utilized for analysis and purification was 5-30% solvent B over 25 minutes with a flow rate of 1 mL/minute at 60 °C. Following purification, the oligonucleotide conjugates were characterized by ESI-HRMS.

Supporting Figures

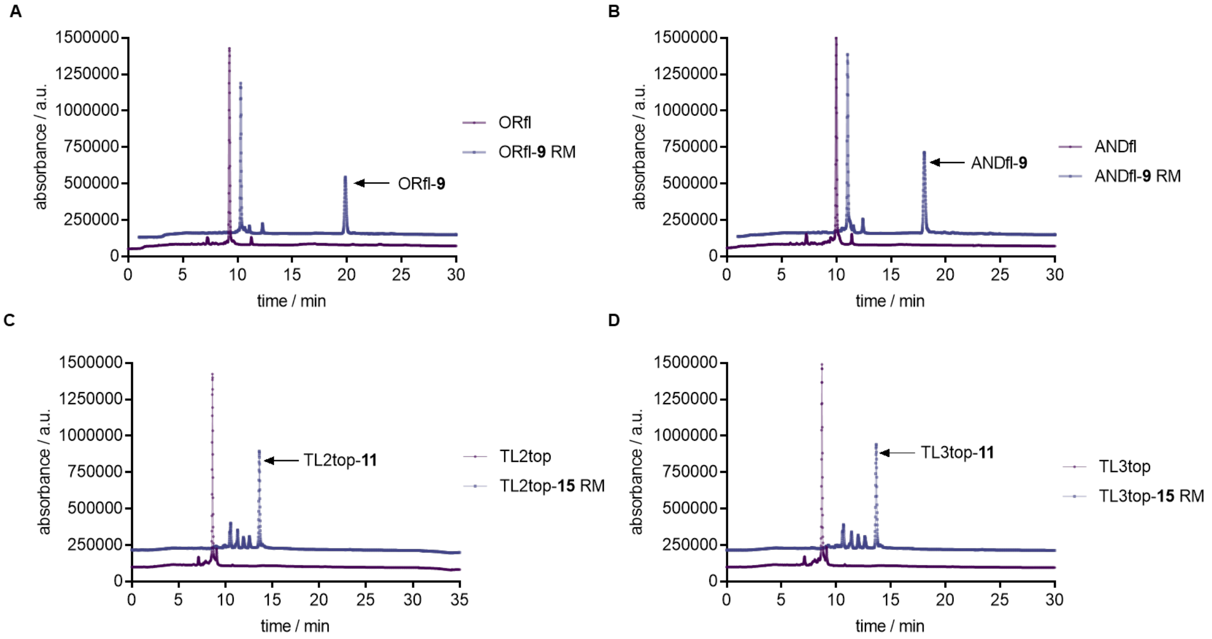

Supporting Figure S1. HPLC chromatograms of the conjugation reaction of A) ORfl-**9**, B) ANDfl-**9**, C) TL2top-**15**, and D) TL3top-**15**. RM stands for reaction mixtures.

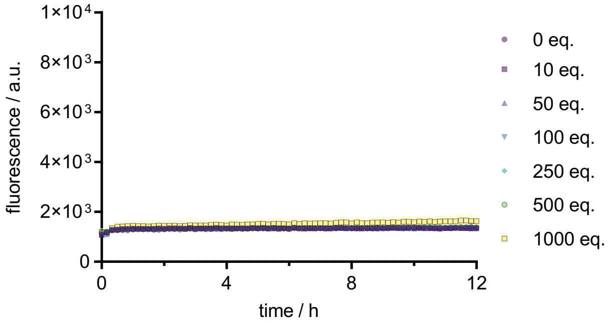

Supporting Figure S2. Reaction of vinyl ether-caged oligonucleotide ONfl-**9** (5'-**9**-ATGTAAATGGTTAGTGATCGA TTGTTATAC-3') with increasing concentrations/equivalents of the methyl tetrazine **10**. ONfl-9 (0.2 µM) was incubated with **10** (2-200 µM) in PBS buffer, pH = 7.4 (30% DMSO), and fluorescence intensity was monitored over 12 h. No fluorophore activation was observed.

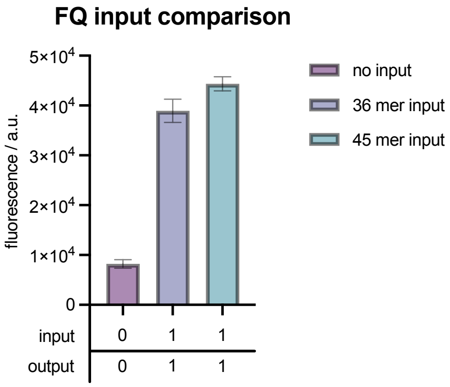

Supporting Figure S3. Activation of FQ reporter in the presence of 1.25 eq of fully complementary 36 mer input or 45 mer input. Mean fluorescence is shown ± s.d. (n=3).

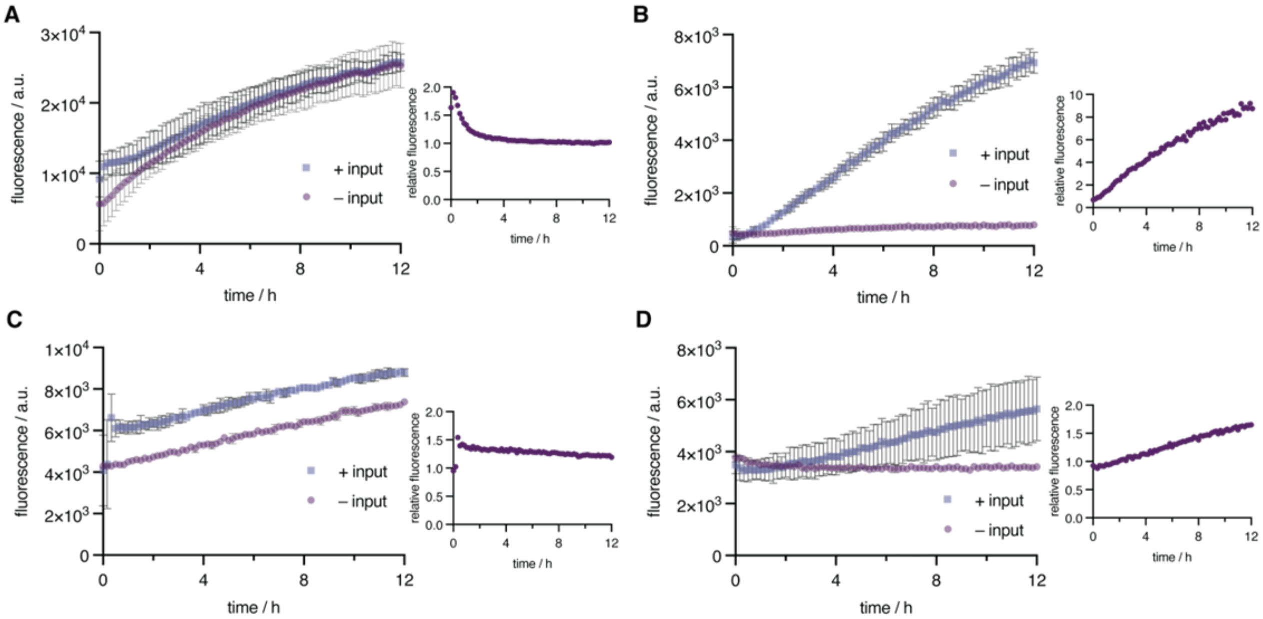

Supporting Figure S4. Activation of IEDDAR in the presence of nucleases. A) FQ and B) IEDDA reporter with and without input DNA were incubated in TE/Mg^{2+} buffer containing 0.02 U of DNase I. C) FQ and D) IEDDA reporter with and without input DNA were incubated in DMEM cell culture media containing 10% [v/v] fetal bovine serum. Mean fluorescence intensity is shown over 12 h (Mean ± s.d.; n = 3). Signal intensity over background is plotted to the right of each time course (relative fluorescence = mean FI+ input / mean FI– input). In contrast to the FQ reporter, the IEDDA-based reporter showed increase in signal despite DNA degradation.

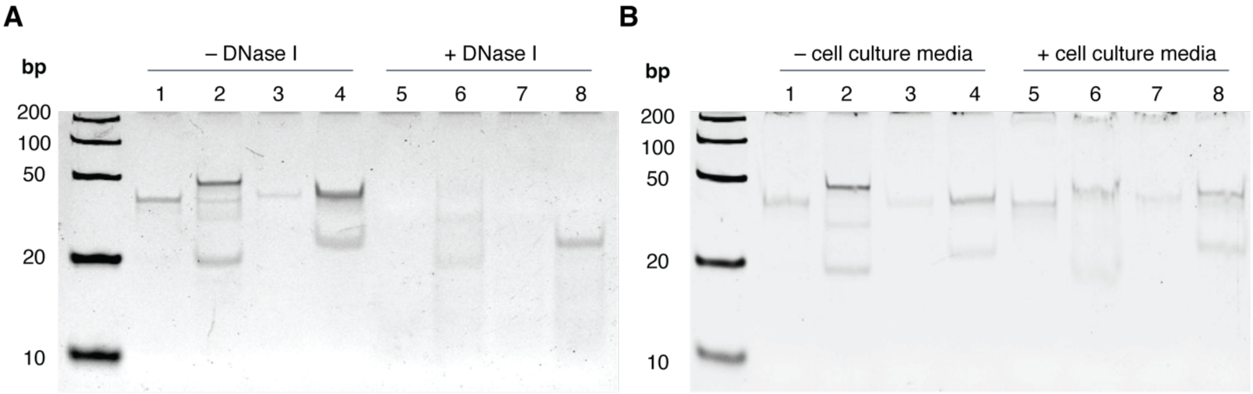

Reporter	Input	Lanes
IEDDA	–	1, 5
	+	2, 6
FQ	–	3, 7
	+	4, 8

Supporting Figure S5. Gel characterization of IEDDAR and FQR strand displacement in the presence of nucleases. FQ and IEDDA reporters with and without input DNA were incubated in TE/Mg^{2+} buffer containing A) 0.02 U of DNase I or B) DMEM cell culture media with 10% [v/v] fetal bovine serum.

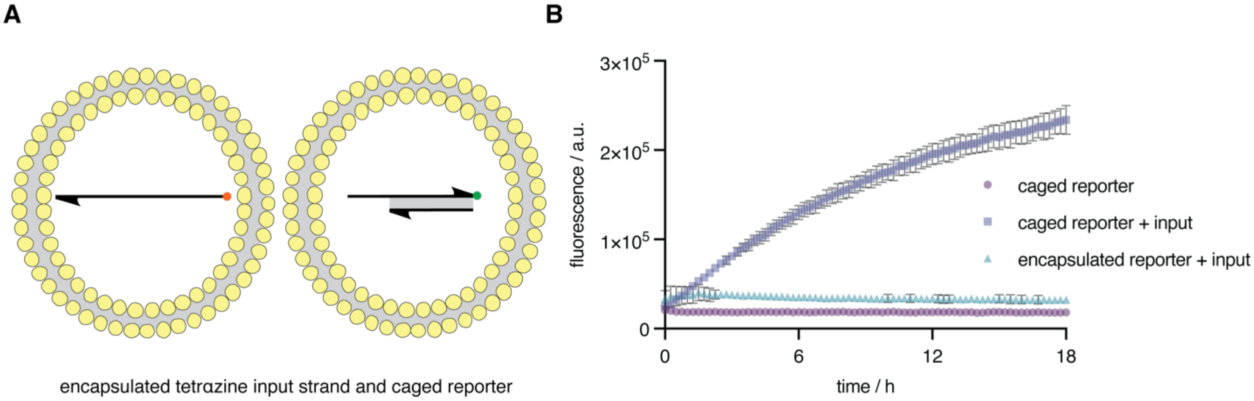

Supporting Figure S6. *In vitro* characterization of reporter input interaction using transfection reagent. A) caged reporter and tetrazine input strand were separately encapsulated in Opti-MEM using FuGENE HD transfection reagent as described in the fluorescence imaging protocol. Following separate incubation, reporter and input were added to a 384-well plate in TE/Mg^{2+} buffer. B) Caged IEDDA reporter with and without input was added to separate wells without transfection reagent as a positive and negative control, respectively. A time course of mean fluorescence intensity is shown over 18 h (Mean ± s.d.; n = 3).

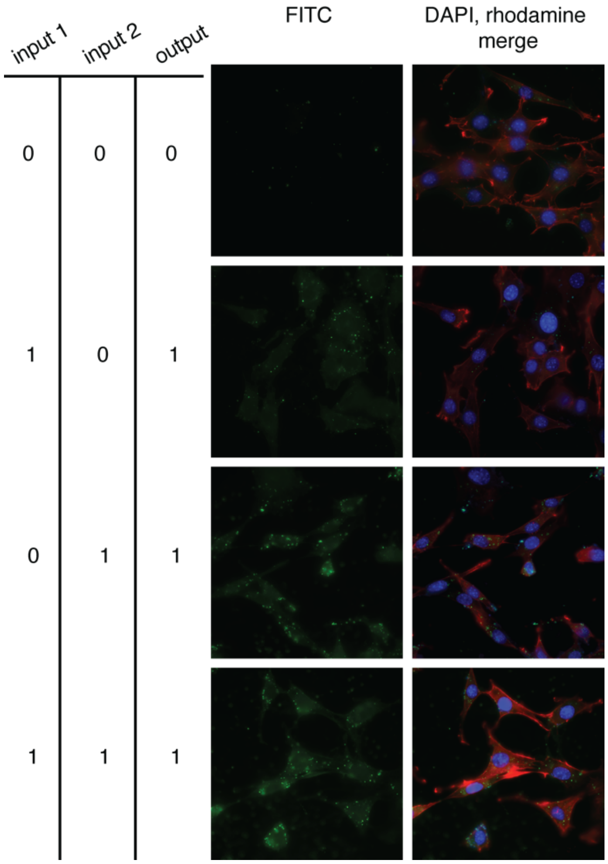

Supporting Figure S7. Templated OR circuit activation in cells. Representative maximum intensity projections of cells transfected with reporter and translator gates and each combination of DNA inputs 1 and 2. Whole cell Z-stack images were acquired using a 63x oil immersion objective at 250 nm intervals in Z-distance.

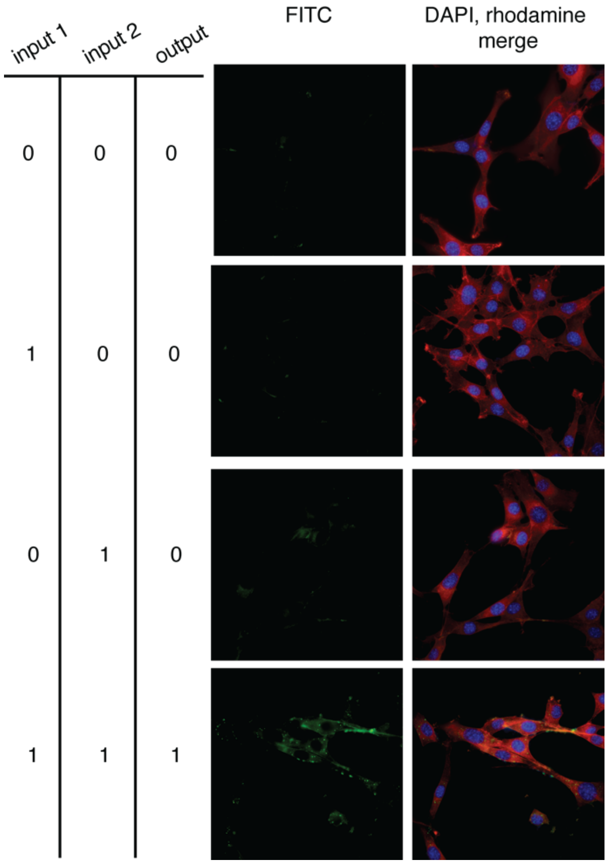

Supporting Figure S8. Templated AND circuit activation in cells. Representative maximum intensity projections of cells transfected with reporter and translator gates and each combination of DNA inputs 1 and 2. Whole cell Z-stack images were acquired using a 63x oil immersion objective at 250 nm intervals in Z-distance.

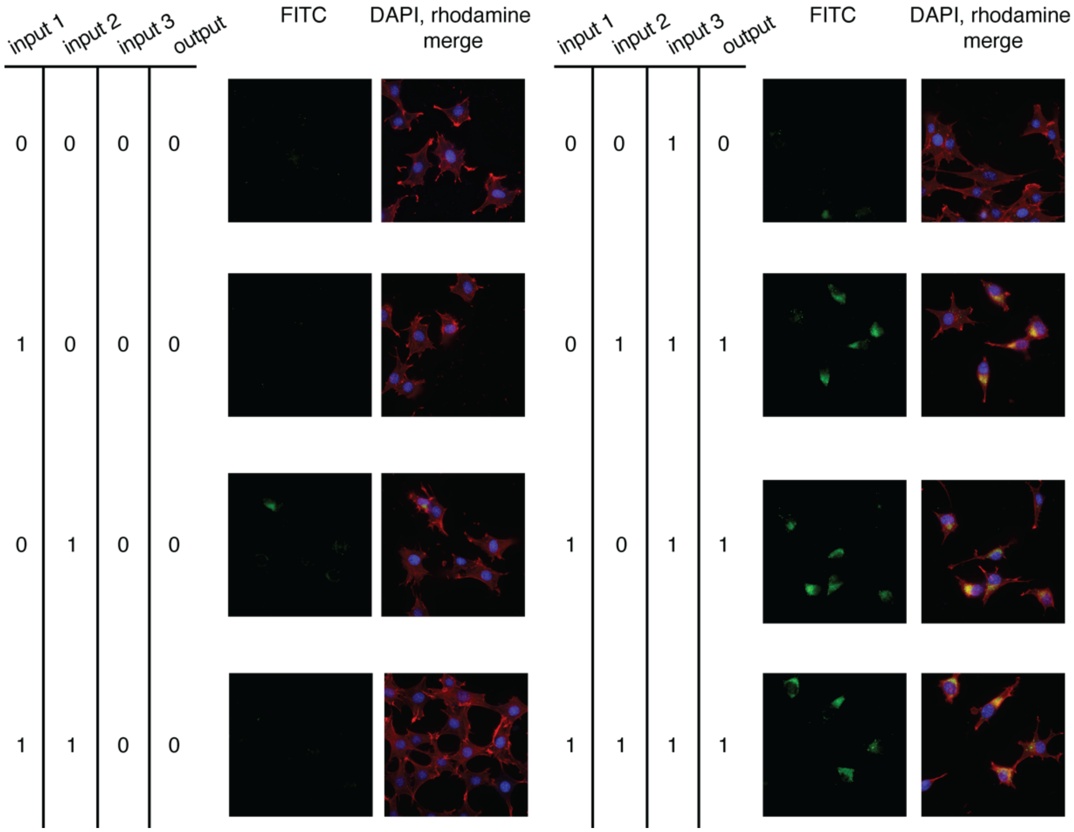

Supporting Figure S9. Templated OR-AND circuit activation in cells. Representative maximum intensity projections of cells transfected with reporter and translator gates and each combination of DNA inputs 1, 2, and 3. Whole cell Z-stack images were acquired using a 63x oil immersion objective at 250 nm intervals in z-distance.

Supporting Table 1.

Circuit(s)	Name	Sequence (5' to 3')
AND, OR-AND	I1	TGAGGTAGTAGGTTGTATGGT
OR	I2	TAAGGCACGCGGTGAATGCC
OR, AND, OR-AND	I3	TAAGGCAGTAGGTTGTATGGT
OR-AND	I4	TGAGATGAAGCACTGTTATGGT
AND, OR-AND	TL1top	AGTAGGTTGTATGGTTGTTTATGTGTTCCCTGATCTTTAGCCTTA
AND, OR-AND	TL1bottom	ACAACCATACAACCTACTACCTCA
OR	TL2top	/5AmMC6/GTTAGATGTTAGTTTCACGAAGACAATGATTAAGGCACGCGGTG
OR	TL2bottom	GGCATTCACCGCGTGCCTTAATC
OR, AND, OR-AND	TL3top	/5AmMC6/GTTAGATGTTAGTTTCACGAAGACAATGATTAAGGCAGTAGGTTG
OR, AND, OR-AND	TL3bottom	ACCATACAACCTACTGCCTTAATC
OR-AND	TL4top	GAAGCACTGTTATGGTTGTTTATGTGTTCCCTGATCTTTAGCCTTA
OR-AND	TL4bottom	ACAACCATAACAGTGCTTCATCTCA
OR	ORds	GTTAGATGTTAGTTTCACGAAGACAATGAT
OR	ORfl	GCCTTAATCATTGTCTTCGTGAAACTAACATCTAAC/3AmMO/
AND, OR-AND	ANDth	TAAGGCTAAAGATCAGGGAACACATAAACAACCATA
AND, OR-AND	ANDds	GTTAGATGTTAGTTTCACGAAGACAATGAT
AND, OR-AND	ANDfl	TGTTTATGTGTTCCCTGATCTTTAGCCTTAATCATTGTCTTCGTGAAACTAAC ATCTAAC/3AmMO/
FQ_rep	Fq	/5IABkFQ/GTTAGATGTTAGTTTCACGAAGACAATGAT
FQ_rep	ORin	GTTAGATGTTAGTTTCACGAAGACAATGATTAAGGC
NA	ONfl	ATGTAAATGGTTAGTGATCGATTGTTATAC

Characterization Spectra

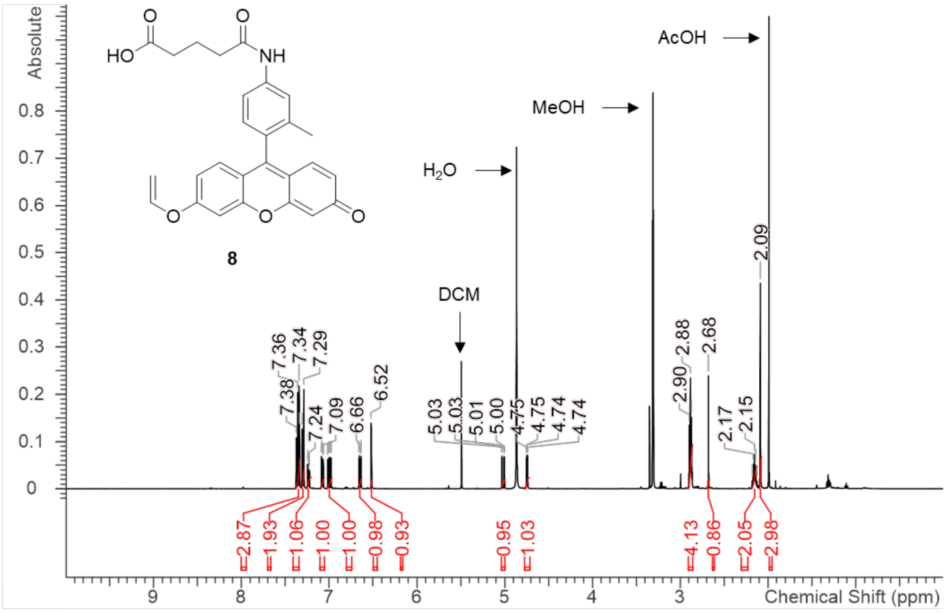

References

(1) Piao, W.; Tsuda, S.; Tanaka, Y.; Maeda, S.; Liu, F.; Takahashi, S.; Kushida, Y.; Komatsu, T.; Ueno, T.; Terai, T.; et al. Development of azo-based fluorescent probes to detect different levels of hypoxia. *Angew Chem Int Ed Engl* **2013**, *52* (49), 13028-13032. DOI: 10.1002/anie.201305784.

(2) Shieh, P.; Hangauer, M. J.; Bertozzi, C. R. Fluorogenic azidofluoresceins for biological imaging. *J Am Chem Soc* **2012**, *134* (42), 17428-17431. DOI: 10.1021/ja308203h.

(3) Wu, H.; Alexander, S. C.; Jin, S.; Devaraj, N. K. A Bioorthogonal Near-Infrared Fluorogenic Probe for mRNA Detection. *J Am Chem Soc* **2016**, *138* (36), 11429-11432. DOI: 10.1021/jacs.6b01625.

(4) Tu, J.; Xu, M.; Parvez, S.; Peterson, R. T.; Franzini, R. M. Bioorthogonal Removal of 3-Isocyanopropyl Groups Enables the Controlled Release of Fluorophores and Drugs in Vivo. *J Am Chem Soc* **2018**, *140* (27), 8410-8414. DOI: 10.1021/jacs.8b05093.
